# Association of anxiety with subcortical amyloidosis in cognitively normal older adults

**DOI:** 10.1038/s41380-018-0214-2

**Published:** 2018-08-16

**Authors:** Bernard J. Hanseeuw, Victoria Jonas, Jonathan Jackson, Rebecca A. Betensky, Dorene M. Rentz, Keith A. Johnson, Reisa A. Sperling, Nancy J. Donovan

**Affiliations:** 1 Department of Neurology, Massachusetts General Hospital, Harvard Medical School, Boston, MA 02114 USA; 2 Department of Radiology, Massachusetts General Hospital, Harvard Medical School, Boston, MA 02114 USA; 3 Department of Neurology, Cliniques Universitaires Saint-Luc, Institute of Neurosciences, Université Catholique de Louvain, Brussels, Belgium; 4 Department of Biostatistics, Harvard T.H. Chan School of Public Health, Boston, MA 02115 USA; 5 Center for Alzheimer Research and Treatment, Brigham and Women’s Hospital, Harvard Medical School, Boston, MA 02115 USA; 6 Department of Neurology, Brigham and Women’s Hospital, Harvard Medical School, Boston, MA 02115 USA; 7 Department of Psychiatry, Brigham and Women’s Hospital, Harvard Medical School, Boston, MA 02115 USA; 8 Department of Psychiatry, Massachusetts General Hospital, Harvard Medical School, Boston, MA 02114 USA

**Keywords:** Psychiatric disorders, Prognostic markers

## Abstract

Late-life anxiety has been associated with increased progression from normal cognition to amnestic MCI, suggesting that anxiety may be a neuropsychiatric symptom of Alzheimer’s disease (AD) pathological changes and a possible marker of anatomical progression in preclinical AD. This study examined whether cortical or subcortical amyloidosis, indicating earlier or later stages of preclinical AD, was associated with greater self-reported anxiety among 118 cognitively normal volunteers, aged 65–90 years, and whether this association was stronger in APOEε4 carriers. Participants underwent Pittsburgh Compound B Positron Emission Tomography (PiB-PET) to assess fibrillar amyloid-β burden in cortical and subcortical regions, and measurement of anxiety using the Hospital Anxiety and Depression Scale-anxiety subscale. Higher PiB-PET measures in the subcortex (striatum, amygdala, and thalamus), but not in the cortex, were associated with greater anxiety, adjusting for demographics, cognition, and depression. Findings were similar using a cortico-striatal staging system and continuous PET measurements. Anxiety was highest in APOEε4 carriers with subcortical amyloidosis. This work supports in vivo staging of amyloid-β deposition in both cortical and subcortical regions as a promising approach to the study of neuropsychiatric symptoms such as anxiety in cognitively normal older individuals. Elevated anxiety symptoms in combination with high-risk biological factors such as APOEε4 and subcortical amyloid-β may identify participants closest to MCI for secondary prevention trials.

## Introduction

Pathological processes impacting late-life anxiety are poorly understood. Approximately 15% of older US adults meet diagnostic criteria for formal anxiety disorders, a lower prevalence compared with younger ages [1], whereas anxiety symptoms in older people appear to be considerably more common. Among participants in a consortium of European studies, anxiety symptoms were endorsed by 32% of non-depressed, community-dwelling older adults and by 87% of those with depression [2], suggesting that etiological factors specific to late-life anxiety may be important but unrecognized.

Precision medicine is a paradigm that promotes the discovery of biologically meaningful disease subtypes through the use of integrated genetic, physiological, and behavioral data [3]. Applying this principle, we sought to define associations of amyloid-β (Aβ), the Alzheimer’s disease (AD) biological marker, and APOEε4, the AD genetic risk factor, with late-life anxiety, in a sample of cognitively normal (CN) older adults. Currently, little is known of the relationship between early neuropsychiatric changes, such as anxiety, and the dynamic process of Aβ deposition in preclinical AD.

Preclinical AD is characterized by progressive Aβ deposition, a process that affects ~25% of CN older adults [4], and occurs for a decade or more before the onset of mild cognitive impairment (MCI) [5]. Expansion of Aβ deposition across brain regions appears to occur in a typical anatomical sequence as described by AD neuropathological studies and staging criteria [6, 7]. Using [^18^F]flutemetamol [8] or [^11^C]PiB [9] positron emission tomography (PET) to detect Aβ plaques in studies of living humans, it is now possible to corroborate neuropathological staging of Aβ progression and to define phenotypic changes that occur during this sequential pathophysiological process [8]. This approach to in vivo staging may allow for the identification of CN older adults with biological markers and observable characteristics of late preclinical AD, a high-value subgroup who may be most likely to benefit from AD-directed therapies. Knowledge of neurobiological mechanisms involved in these observed clinical changes, such as anxiety, may also reveal novel disease-modifying strategies.

Aβ PET studies have commonly used a binary approach to classify individuals as Aβ positive or negative based on tracer uptake in neocortical regions. In recent work using longitudinal Aβ PET data from Alzheimer’s Disease Neuroimaging Initiative (ADNI) and the Harvard Aging Brain Study (HABS), we established a three-stage Aβ framework that classifies participants as high or low Aβ in neocortical and in striatal (caudate and putamen) aggregates. We observed that striatal Aβ deposition (stage 2) occurs after neocortical Aβ (stage 1) during disease progression, and that individuals with high striatal Aβ showed more rapid cognitive decline, even within the CN group [10].

Using this new in vivo Aβ staging framework, we aimed to examine cross-sectional associations of cortical and striatal Aβ with self-reported anxiety in CN older adults. Supported by human and animal research implicating striatal and other subcortical structures and circuits in instinctual and learned emotional responses, including anxiety [11, 12] and depression [13–16], we hypothesized that anxiety may be a symptom of late preclinical AD and would be elevated in association with stage 2 but not a stage 1 Aβ deposition. We further hypothesized that this association would be independent of age, sex, cognitive reserve, global cognition, and depression, and would be stronger in carriers of the APOEε4 allele [17].

## MATERIALS AND METHODS

### Participants

The sample comprised 118 English-speaking, community-dwelling older men and women participating in HABS. They were all CN based on a global Clinical Dementia Rating = 0 [18], Mini-Mental State Examination (MMSE) ≥ 26 [19], and education-adjusted norms on the Logical Memory delayed recall [20]. All clinical and imaging data were obtained during the fourth annual study visit. At initial screening, all included participants were free from active, serious medical, or neurological conditions. Individuals with a history of schizophrenia, schizoaffective disorder, bipolar disorder, moderate or severe major depressive disorder, or active substance-use disorder were excluded. A history of depression adequately treated with antidepressant medication was allowed. At screening, all participants scored below 11 on the 30-item Geriatric Depression Scale (GDS) [21]. No psychiatric or GDS exclusions were applied at subsequent study visits. Research protocols were approved by the Partners Human Research Committee. All participants provided informed consent.

### Clinical measures

Anxiety was measured by self-report using the total anxiety-subscale score from the 14-item Hospital Anxiety and Depression Scale [22] (HADS-anxiety; each of the 7 anxiety symptoms was rated for frequency, range 0–3, with higher score indicating greater anxiety; total score range 0–21). Anxiety ratings were completed in a blinded manner with regard to other assessments and procedures. Self-reported depression was calculated as the total score from the 30-item GDS (item score 0–1, total score 0–30, greater score indicates greater severity) [21]. Based on APOEε4 genotype, participants were classified as either APOEε4 carriers or non-carriers.

### Imaging

PET scanning was conducted using a Siemens HR+ scanner (three-dimensional mode; 63 images; 15.2 cm axial field-of-view; 5.6 mm transaxial resolution and 2.4 mm slice interval). After attenuation and motion correction, distribution volume ratio (DVR) images were created with Logan plotting (40–60 min interval, gray matter cerebellar reference region). Data were co-registered to each participant’s magnetic resonance imaging and anatomically parcellated using Freesurfer v5.1. Partial volume correction was conducted using geometric transfer matrix.

Pittsburgh Compound B (PiB) signal was measured in neocortical and subcortical aggregates. The neocortical aggregate included frontal, lateral temporal, parietal, and retrosplenial regions-of-interest. The striatal aggregate included putamen and caudate. Neocortical and striatal thresholds for PiB were defined using Gaussian mixture models in the entire HABS cohort (*n* = 279) as previously reported [10]. These thresholds were then used to define three PiB stages: PiB stage 0 included participants who had low PiB in both the neocortex and striatum. PiB stage 1 included participants who had high neocortical but low striatal PiB. PiB stage 2 included participants with high PiB in both the neocortex and striatum [10].

All participants with high striatal PiB also had high neocortical PiB. Accordingly, participants in stage 0 were all low in neocortical PiB (PiB−) and all participants in PiB stages 1 and 2 were high in neocortical PiB (PiB+), based on binary neocortical PiB groups.

### Statistical analyses

Unadjusted comparisons of clinical variables according to PiB stages were performed using linear regressions.

Three series of multiple linear regression models with HADS-anxiety as the dependent variable were fit for the purpose of estimating associations of PiB variables and APOEε4 with HADS-anxiety scores. All models controlled for age, sex, education, MMSE, and GDS. *P*-values were two-sided. HADS-anxiety scores adjusted for covariates were normally distributed (Lilliefors test = 0.068, criterion value for non-normal distribution = 0.082, *p* = 0.19).

In the first series of models using a standard binary PiB classification (reference PiB−), we fit three distinct models in which we estimated the associations of HADS-anxiety with neocortical the PiB+/PiB− group, with APOEε4 carrier status, and with PiB+/PiB− group modified by APOEε4 status. We fit an analogous series of models (series 2) using the three-stage PiB classification (reference stage 0).

In a third series of models, we examined continuous measures of neocortical or striatal PiB as predictors of HADS-anxiety in separate models. To determine whether associations of PiB with HADS-anxiety were specific to striatum or were observed in other subcortical regions, we also examined continuous measures of amygdala and thalamus PiB as predictors of HADS-anxiety in adjusted models. These final four analyses were carried out using data from all participants classified as neocortical PiB+.

Analyses were performed using Matlab v9.0.

## RESULTS

Demographic, genetic, and clinical data are shown for the sample and by PiB stages 0–2 in Table 1. There were no significant differences in sex or education across PiB stages. Mean age was numerically higher in stages 1 and 2 compared with stage 0 and was significantly different between stage 1 and stage 0. There was a significantly higher proportion of APOEε4 carriers in stage 1 and 2 compared with stage 0 (Table 1). Stage 1 and stage 2 did not differ in demographic characteristics or APOEε4 carrier status. Only one participant was homozygous for the APOEε4 allele (and was classified as stage 1). Mean HADS-anxiety score for the sample was 3.8 (range 0–12; possible range 0–21) and mean GDS score was 3.6 (range 0–17; possible range 0–30). Eleven participants (9.3% of the sample) scored above cut-off for clinically significant anxiety (> 7) and seven participants (5.9% of the sample) scored above cut-off for mild depression based on the GDS (> 10). Only one participant scored above both anxiety and depression cut-offs. GDS scores did not differ across PiB stages, as demonstrated by a linear regression predicting GDS, with or without adjustment for age, sex, education, and MMSE (all *β* ≤ 1.3, *p* > 0.10).

**Table 1:** Demographic and clinical data for study participants

	Whole sample	PiB stage 0 low neocortical, low striatal	PiB stage 1 high neocortical, low striatal	PiB stage 2 high neocortical, high striatal
***N***	**118**	**75**	**28**	**15**
Age, years	75.9 (5.7)	75.0 (5.6)	77.8 (5.6)*	77.4 (5.7)
Education, years	16.1 (2.7)	16.3 (2.8)	15.6 (2.7)	16.4 (2.7)
Female, % *(N)*	61.9 (73)	58.7 (44)	67.9 (18)	66.7 (10)
E4 carriers, % *(N)*	28.0 (33)	13.3 (10)	53.6 (15)*	53.5(8)*
MMSE (0–30)	29.2 (1.1)	29.2 (1.1)	28.8 (1.1)	29.5 (0.8)
Geriatric Depression Scale (0–30)	3.6 (3.4)	3.3 (3.2)	4.5 (4.0)	2.8 (2.0)
HADS-Anxiety Subscale (0–21)	3.8 (2.9)	3.7 (2.7)	3.6 (3.1)	4.9 (3.9)*

*Significantly different from individuals with PiB Stage 0 (*p* < 0.05) by linear regression. Unless otherwise indicated, data are reported as mean (SD) values. *HADS* Hospital Anxiety and Depression Scale, *MMSE* Mini-Mental State Examination, *PiB* Pittsburgh Compound B

**Fig. 1:**
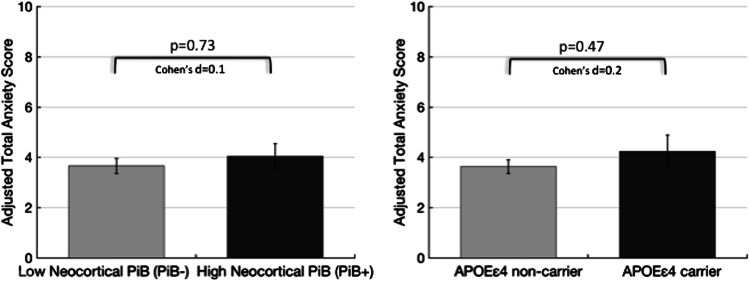
Anxiety scores do not differ by Neocortical PiB+/− Group or APOEε4 carrier status. Anxiety scores adjusted for age, sex, education, and Mini-Mental State Examination and Geriatric Depression Scale scores are shown. Error bars are SEM. Cohen’s *d* effect size (small 0.2, medium 0.4, large 1.0)

In series 1 models, the mean HADS-anxiety score did not differ between binary neocortical PiB− and PiB+ groups (Table 2; Fig. 1 left) or between APOEε4 carriers and non-carriers (Table 2; Fig. 1 right), adjusting for age, sex, education, MMSE, and GDS. Neither age, education, nor MMSE was associated with HADS-anxiety in these models. HADS-anxiety was significantly associated with female sex such that the mean anxiety score was 1.31 points higher in women than in men, adjusted for other covariates (95% confidence interval (CI), 0.37–2.45, *p* = 0.009). Mean HADS-anxiety score also increased 0.23 points for each additional point of GDS (95% CI, 0.10–0.40, *p* = 0.001). The interaction of neocortical PiB+ group and APOEε4 was marginally but not significantly associated with higher HADS-anxiety (Table 2).

**Table 2:** Estimates for PiB-PET variables and APOEε4 carrier status predicting HADS-anxiety subscale scores in serial multivariate models

Predictor	Unstandardized *β*-estimate (SE)	95% CI	Model *R*^2^	*P*-value
Series 1 (*n* = 118): neocortical PiB+/PiB− groups and APOEε4 carrier status				
PiB+ group (reference PiB−)	+ 0.2 (0.5)	− 0.9 to 1.3	0.16	0.73
APOEε4 carrier (reference non-carrier)	+ 0.4 (0.6)	− 0.7 to 1.5	0.16	0.47
PiB+ group * APOEε4 carrier	+ 2.3 (1.3)	− 0.3 to 4.9	0.19	0.07
Series 2 (*n* = 118): PiB stages (reference PiB stage 0) and APOEε4 carrier status				
PiB Stage 1	− 0.5 (0.6)	− 1.7 to 0.8	0.19	0.46
PiB Stage 2	+ 1.8 (0.9)	0.1 to 3.6		0.04
PiB Stage 1 * APOEε4 carrier	+ 1.2 (1.4)	− 1.5 to 3.9	0.24	0.38
PiB Stage 2 * APOEε4 carrier	+ 4.3 (1.7)	1.0 to 7.6		0.01
Series 3 (*n* = 43): continuous PiB measures in neocortical PiB+ participants				
Neocortical PiB	+ 0.6 (1.0)	− 1.4 to 2.6	0.20	0.54
Striatal PiB	+ 3.0 (1.6)	− 0.1 to 6.1	0.26	0.07
Thalamus PiB	+ 6.7 (2.5)	1.7 to 11.7	0.32	0.01
Amygdala PiB	+ 9.5 (4.0)	1.6 to 17.3	0.29	0.02

Each row shows results for the predictor or predictors of interest in separate models. All models are adjusted for age, sex, education, MMSE, and GDS scores. PiB Stage 0 (low neocortical, low striatal PiB). PiB Stage 1 (high neocortical, low striatal PiB). PiB Stage 2 (high neocortical, high striatal PiB). *CI* confidence interval, *GDS* Geriatric Depression Scale, *HADS-anxiety* Hospital Anxiety and Depression Scale, *MMSE* Mini-Mental State Examination, *PiB* Pittsburgh Compound B

**Fig. 2:**
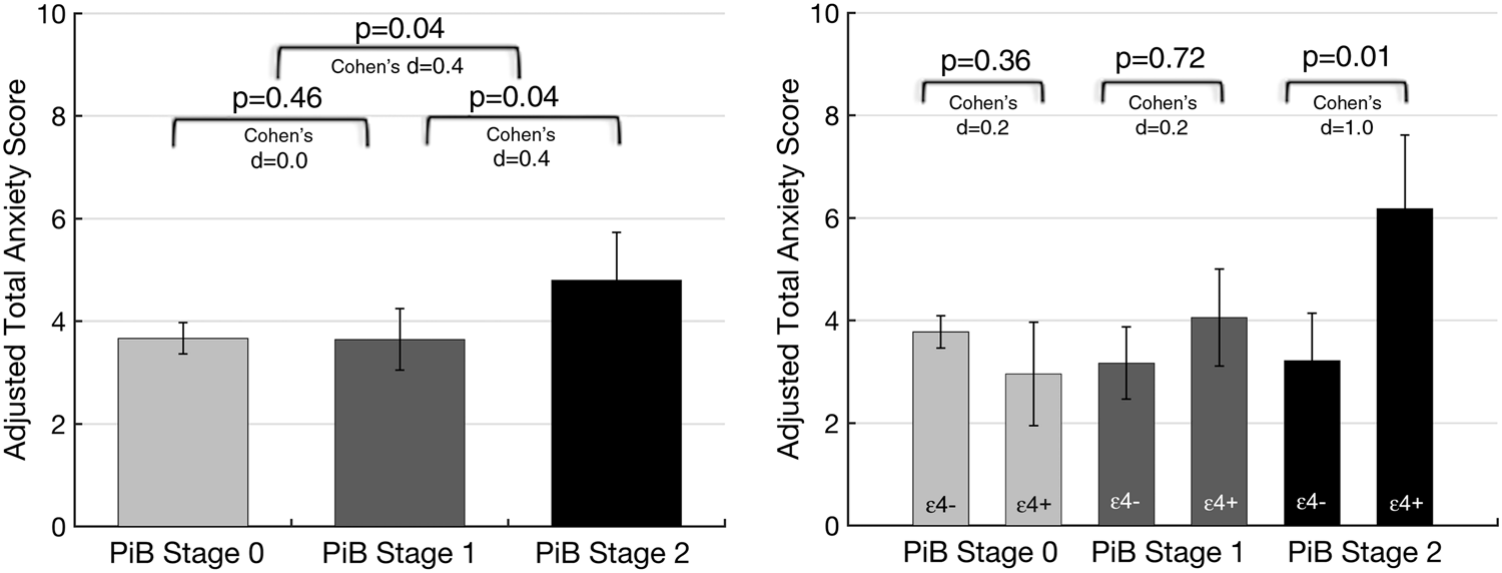
Anxiety scores are higher in PiB stage 2, specifically in APOEε4 carriers. Anxiety scores adjusted for age, sex, education, and Mini-Mental State Examination and Geriatric Depression Scale scores are shown. Error bars are SEM. Cohen’s *d* effect size (small 0.2, medium 0.4, large 1.0)

In series 2 models, PiB stage 2 compared with stage 0 was associated with higher HADS-anxiety (Table 2; Fig. 2 left). The mean HADS-anxiety score was 2 points higher for PiB stage 2 compared with PiB stage 0 participants, adjusted for other covariates. The PiB stage 2-APOEε4 interaction term was also significantly associated with greater HADS-anxiety (Table 2). Notably, the association of PiB stage 2 with HADS-anxiety was observed specifically in APOEε4 carriers compared with non-carriers (Fig. 2 right). There was no significant difference in HADS-anxiety between PiB stage 1 and stage 0 (Table 2). The interaction of PiB stage 1 and APOEε4 was also not significantly associated with HADS-anxiety. GDS and female sex, but not with the other covariates, were associated with HADS-anxiety in these models (for GDS, *β* = 0.27, 95% CI, 0.13–0.42, *p* = 0.001; for female sex, *β* = 1.40, 95% CI, 0.40–2.40, *p* = 0.008).

Simple examination of the 11 participants with anxiety scores above cut-off for clinically significant anxiety exemplifies these statistical associations. Six out of 75 participants in the neocortical PiB− group had high anxiety compared with 5 out of 43 in the neocortical PiB+ group. Among the six PiB− participants with high anxiety, one was a APOEε4 carrier, whereas all five PiB+ participants with high anxiety were APOEε4 carriers. Within the PiB+ group, one participant was PiB Stage 1 and was depressed (GDS > 10), and the remaining four participants were PiB Stage 2 and not depressed.

**Fig. 3:**
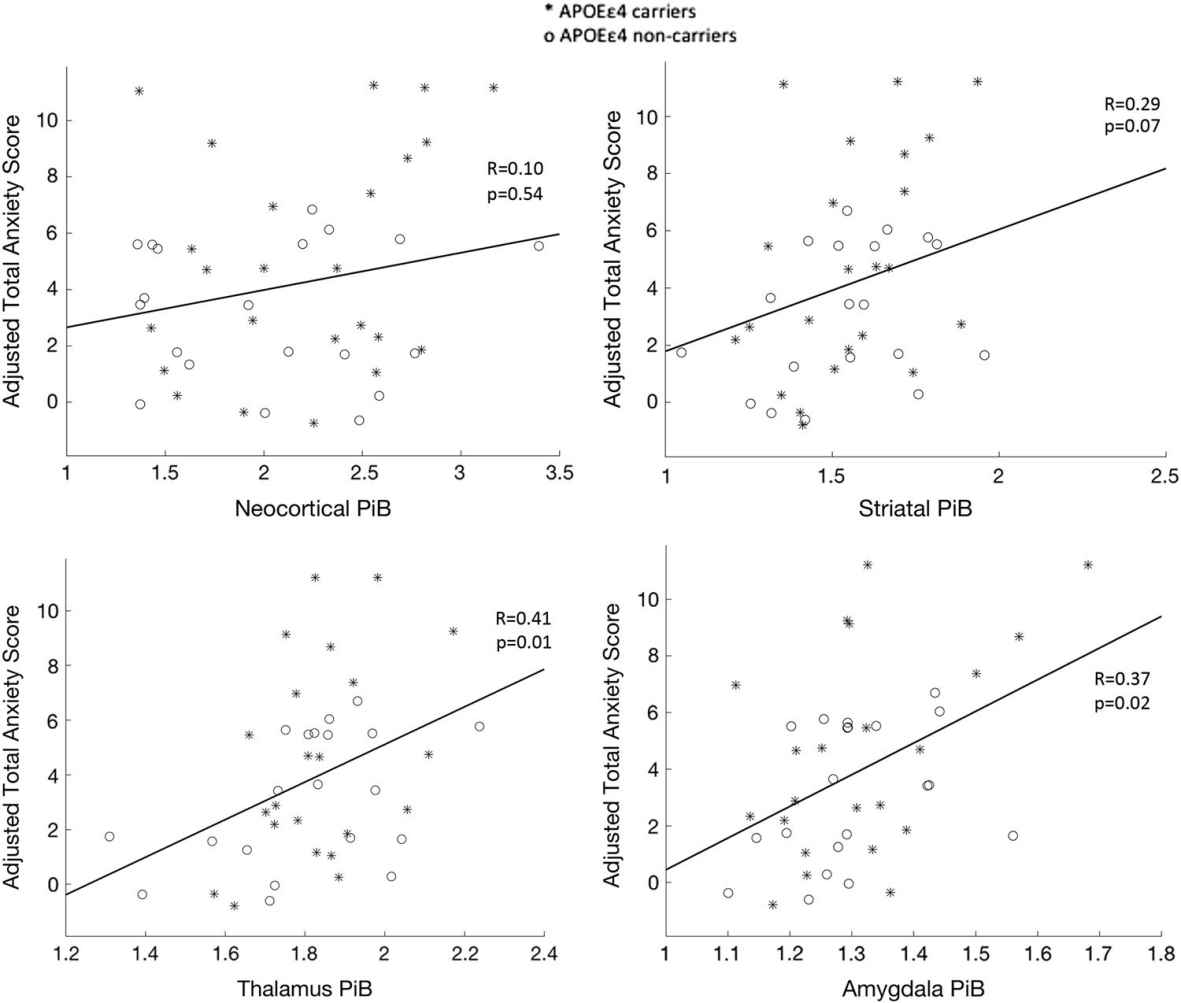
Among cognitively normal older adults with elevated neocortical PiB binding, anxiety scores increase as a function of subcortical PiB binding. Regression lines for anxiety and PiB binding in cortex and selected subcortical regions are shown. Anxiety scores are adjusted for age, sex, education, Mini-Mental State Examination and Geriatric Depression Scale scores. Partial Pearson’s correlations (*R*) between PiB binding and anxiety scores, adjusted for covariates are shown

In the final four models examining continuous measures of PiB, greater striatal PiB was marginally associated with higher HADS-anxiety, whereas the association of continuous neocortical PiB with HADS-anxiety was nonsignificant, adjusting for all covariates (Table 2; Fig. 3). Higher HADS-anxiety was significantly associated with greater PiB uptake in other subcortical regions, specifically, the thalamus and amygdala (Table 2; Fig. 3). After adjusting for other covariates, mean HADS-anxiety was 0.3 points higher for each additional 0.1 PiB DVR for the striatum, 0.7 points higher per 0.1 DVR for the thalamus, and 1 point higher per 0.1 DVR for the amygdala. Adjusting for APOEε4 did not modify these associations. Results for all Series 1–3 models were similar with and without adjusting for GDS and sex.

## DISCUSSION

We evaluated anxiety symptoms in CN older adults who were classified according to a new three-stage model of preclinical AD progression based on regional Aβ deposition and found that participants with advanced amyloidosis, indicated by high neocortical and high striatal Aβ (stage 2), had significantly greater anxiety than those with low neocortical and low striatal Aβ (stage 0). This effect was moderately strong. By comparison, anxiety ratings did not differ between individuals with high neocortical but low striatal Aβ (stage 1) and those with low Aβ in both neocortex and striatum Aβ (stage 0). These results suggest that anxiety symptoms may signal Aβ expansion beyond the neocortex and serve as a marker of pathologic progression typical of early AD. As these associations were independent of depressive symptom burden, anxiety symptoms may have specific prognostic implications, compared with other depression-related symptoms, in preclinical AD.

Our findings also indicate that the APOEε4 allele is an important modifying factor in the pathogenesis of anxiety, in addition to Aβ stage. The association of PiB stage 2 with HADS-anxiety was observed specifically in APOEε4 carriers. Brain Aβ burden and APOEε4 genotype appear to be interacting biological factors associated with greater late-life anxiety in CN older people.

From a precision medicine perspective, our findings suggest that a subset of CN individuals with late-life anxiety may be at high risk of cognitive impairment due to AD and could be identified by APOEε4 genotype and Aβ-PET staging. These diagnostic tests may become important for geriatric psychiatrists and other specialists to recognize and refer patients for AD-directed interventions or to indicate specific anxiety treatments.

These results extend prior research that provided initial evidence for anxiety as a neuropsychiatric manifestation of AD at the stage of MCI. Using combined cross-sectional data from the ADNI and the Development of Screening Guidelines and Criteria for pre-Dementia Alzheimer’s Disease study, Ramakers et al. [23] found that informant-based measures of anxiety, irritability, and agitation, but none of nine other Neuropsychiatric Inventory items, were associated with abnormal cerebrospinal fluid (CSF) concentrations of amyloid-Aβ_1−42_ in older adults with MCI. Anxiety alone was associated with elevated CSF total tau, a neurodegeneration marker, consistent with anxiety as a stronger marker of AD pathologies and prodromal AD, compared with a range of other NPS [23].

Prior research further suggests that anxiety may be elevated in a subgroup of CN elderly during preclinical AD. In a study from the Australian Imaging, Biomarkers and Lifestyle (AIBL) group, Holmes et al. [17] found that CN APOEε4 carriers were more likely to report high anxiety than non-carriers, a small cross-sectional effect that was potentiated by Aβ+ group and female sex (adjusting for age and education). Using a standard binary approach to Aβ-PET classification, a main effect of Aβ+ group with anxiety was not present, in line with our results which found no significant main effect of neocortical Aβ burden with anxiety. Although the AIBL study found a significant positive association of the APOEε4-by-Aβ+ group interaction with greater anxiety, we found a marginally significant association in an analogous model, possibly attributable to the smaller size of this sample. Comparison of these studies highlights the potential advantage of the three-stage PiB framework. Despite the smaller sample size, our results suggest that greater anxiety among Aβ+, APOE ε4 carriers may be associated with high striatal Aβ, a smaller and more specific subset of CN individuals than those who would be defined as Aβ+ by a typical binary approach.

Other observational studies have revealed no significant association or a weak association of PET-derived measures of neocortical Aβ with anxiety and depressive symptoms in CN community-dwelling older adults. Krell-Roesch et al. [24] examined cross-sectional data from over 1000 participants in the Mayo Clinic Study of Aging and found that each one point increase in the Beck Anxiety Inventory-II score was associated with a slightly, higher odds of being in a binary Aβ+ compared with the Aβ− group [odds ratio 1.03 (1.01–1.08)], adjusted for age and sex. The association of Beck Depression Inventory-II Scores and the Aβ+ group was marginally significant [24].

Additional studies from the AIBL group and the HABS found no cross-sectional association of neocortical Aβ+ group or continuous measures of neocortical Aβ with depression measured by the GDS scale [25, 26]. In longitudinal analyses, both studies found that baseline measures of neocortical Aβ predicted rising anxiety or anxiety–apathy symptom cluster scores over time, although effects were small. Collectively, these three large cohort studies point to weak associations of anxiety symptoms with brain Aβ that are difficult to detect using standard neocortical aggregate Aβ-PET measures.

Our findings support Aβ-PET staging as a promising approach to the study of neuropsychiatric symptoms in preclinical AD, which builds upon established neuropathological staging criteria for AD. Aβ-PET has been well validated as a measure of neocortical and striatal plaque burden in prior studies [10, 27].

Neuropathological studies have found that Aβ deposition in the form of senile plaques begins in the neocortex (phase 1), followed by deposition within entorhinal, hippocampal, and amygdalar regions (phase 2), then in subcortical structures including the striatum (phase 3), the thalamus (phase 4), the brainstem, and finally the cerebellum (phase 5) [6]. Striatal Aβ plaque density is correlated with tau-related neurodegeneration and neurofibrillary tangle stage, consistent with its status as a marker of advanced Aβ deposition and disease progression [28]. Thus, participants with striatal amyloidosis, defined by Aβ-PET, could be experiencing higher anxiety as a symptom of Aβ or tau-related alterations in neuronal activity in medial temporal, limbic subcortical, or striatal regions.

In the final series of models, rather than using a staging approach, we explored associations of anxiety with continuous, regional measures of subcortical Aβ in the subset of participants, classified typically, as Aβ+. Although studies of late-life anxiety and striatum are lacking, the caudate and putamen have been implicated in structural imaging studies of late-life depression [16, 29] and in major depressive disorder in mixed age samples [30–33]. Recently, intrinsic inter-network connectivity abnormalities have been correlated with depression and anxiety severity in older adults with late-life depression [34]. Reported findings involved the right executive control network and subcortical (thalamus, basal ganglia, and ventral striatum) networks [34]. Importantly, amygdala network dysfunction has also been described in late-life depression with and without MCI, supporting the amygdala as a key emotional processing network hub in older adults [15].

Using the smaller subset of participants with high neocortical Aβ, we found moderately strong and significant associations of anxiety with continuous measures of Aβ in the thalamus and amygdala, but not with neocortical Aβ. Although these findings support the association of anxiety with subcortical amyloidosis, they should be interpreted with caution. High nonspecific uptake of PIB in the amygdala and thalamus has been observed and off-target binding may be contributing to these effects. In addition, the association of anxiety with continuous striatal Aβ measures was only marginally significant in this small sample and is not significant after correction for multiple comparisons. Further analyses are needed in larger samples using Aβ and tau-PET to extend these analyses.

Late-life depression and anxiety symptoms have been associated with hypermetabolic activity in networks comprising subcortical and medial temporal structures [35, 36]. Leal et al. [35] described increased amgydala– entorhinal–hippocampal network activity during an emotional memory task in CN older adults with elevated depression and anxiety symptoms, compared with those without, noting that this network is vulnerable to a wide range of age-related pathologies, including preclinical AD. In other work describing regional hyperactivity in late-life depression, Diaconescu et al. [36] reported Citalopram-related reductions in glucose metabolism and anterior cingulate-seed based connectivity within a subcortical–limbic–frontal network, effects that were also correlated with improvements in anxiety and depression.

These preliminary findings, informed by research in non-geriatric samples, suggest a model in which AD-related alterations within anxiety modulating circuits may be associated with heightened tonic anxiety, increased glutaminergic neurotransmission from prefrontal regions to the amygdala and greater local Aβ production [37, 38]. In that regard, selective serotonin-reuptake inhibitor use, or other anxiolytic medications, may mitigate these pathophysiological effects and could have modifying effects on AD progression in certain CN subgroups [36–39]. Specifically, Burke et al. [40] analyzed National Alzheimer’s Coordinating Center data for more than 12,000 CN older adults and found that participants with anxiety who were prescribed anxiolytic medications were less likely to progress to MCI than those with anxiety and no anxiolytic medication use over a 4-year period.

Limitations to this study are noted. As anxiety and depression scores for the sample were mostly in the subthreshold range, we have not established whether the observed relationship of Aβ to anxiety is present across the full range of anxiety scores. Additional analyses examining a broader range of anxiety scores would be important to extend and translate these findings. These analyses adjusted for depression and female sex, established predictors of late-life anxiety in high-functioning older individuals [17, 41], but we did not explore potential confounders such as subjective cognitive concerns or subtle objective cognitive declines [42]. We also did not consider possible syndemic neuroinflammatory effects, such as activation of glia and other innate immune mechanisms, which may be involved in anxiety and early AD pathologies [43–45]. Our approach, using both cortical and subcortical Aβ PET data, allows for more specific characterization of the regional expansion of Aβ pathology; however, this approach reduces statistical power, as it decreases the number of individuals in each subgroup. Our findings were based on a limited number of individuals with anxiety and subcortical amyloidosis and should be replicated in larger samples. Although our findings provide indirect evidence that anxiety is an outcome of Aβ accumulation, bidirectional and longitudinal analyses are necessary to define these causal relationships, the role of related pathologies such as soluble Aβ and tau, and possible involvement of neuroinflammatory processes, in the pathogenesis of anxiety symptoms during preclinical AD. Indeed, as striatal Aβ plaque density is correlated with tau-related neurodegeneration and neurofibrillary tangle stage, anxiety symptoms within this framework may point to overall pathologic load and biologic progression in early AD.

In conclusion, we found that CN older people with more advanced amyloidosis, as indicated by high striatal Aβ, reported significantly more anxiety than those with low Aβ or high cortical Aβ alone, and this difference was strongest in APOEε4 carriers. These findings demonstrate the potential utility of genetic testing and Aβ-PET imaging in the assessment of late-life anxiety and further substantiate the role of anxiety as a behavioral marker in late preclinical AD. Recognition of anxiety symptoms in combination with biological factors such as APOEε4 or advanced Aβ stage may enhance the identification of older individuals at high risk for progression to MCI or AD dementia.
